# Using sedimentary ancient DNA in coastal and marine contexts to explore past human–environmental interactions in Australia

**DOI:** 10.1098/rstb.2024.0032

**Published:** 2025-07-10

**Authors:** Matthew A. Campbell, Ingrid Ward, Alison Blyth, Morten E. Allentoft

**Affiliations:** ^1^ Trace and Environmental DNA (TrEnD) Lab, School of Molecular and Life Sciences, Curtin University, Perth, Australia; ^2^ School of Biological Sciences, The University of Western Australia, Perth, Australia; ^3^ School of Molecular and Life Sciences, Curtin University, Perth, Australia; ^4^ School of Earth and Planetary Sciences, Curtin University, Perth, Australia; ^5^ Section for GeoGenetics, Globe Institute, University of Copenhagen, Copenhagen, Denmark

**Keywords:** sea-level rise, sedimentary ancient DNA, ecological change, human–environmental interactions

## Abstract

Over the 65 000 years of human occupation in Australia, sea levels have fluctuated significantly, notably rising from −120 m around 21 000 years ago, submerging vast areas of the continental shelf. Current coastal ecosystems stabilized about 5000 years ago, leaving many early cultural landscapes underwater, complicating the study of ancient human activity. Sedimentary ancient DNA (sedaDNA) analysis, a powerful tool for monitoring ecological changes and human–environment interactions, has recently gained attention but its exploration is still in its early stages in Australia. This approach holds great potential for investigating shifts in resource and land-use changes, the introduction of non-native species and distinguishing between human and natural impacts on biodiversity. Despite challenges with DNA preservation due to Australia’s harsh climate, organic-rich coastal and marine sediments may provide favourable conditions for sedaDNA. We review case studies across Australia, showcasing how sedaDNA offers valuable insights into past coastal ecologies and can contribute to developing a sustainable biocultural landscape.

This article is part of the theme issue ‘Shifting seas: understanding deep-time human impacts on marine ecosystems’.

## 1. Introduction

The history of human occupation in Australia spans tens of thousands of years. Archaeological evidence indicates that the presence of humans on the Australian mainland extends back at least 65 000 years [1,2], predating modern human occupation in Europe and the Americas [3]. Indigenous Australians exhibited a high level of adaptability, thriving in a diverse range of environments, from lush coastal regions to arid interiors [4]. Indigenous knowledge of the land, waters and their resources is sophisticated, with evidence showing that plant production and biodiversity were increased through practices such as seed dispersal, soil turnover and controlled burning [5,6]. Furthermore, it has been argued that they maintained a symbiotic relationship with their environment, practising sustainable methods that contributed to preserving the delicate equilibrium of local ecosystems [7–9]. Others have argued for more prominent anthropogenic impacts. For example, early human habitation in Australia also coincided with the decline of the megafauna, leading to an extensive and ongoing academic debate as to the weighting of anthropogenic and climatic factors in this extinction (e.g. [10,11]). Regardless of the underlying cause, the loss of keystone species would have resulted in further significant changes to the ecosystem [12–14].

**Figure 1.**
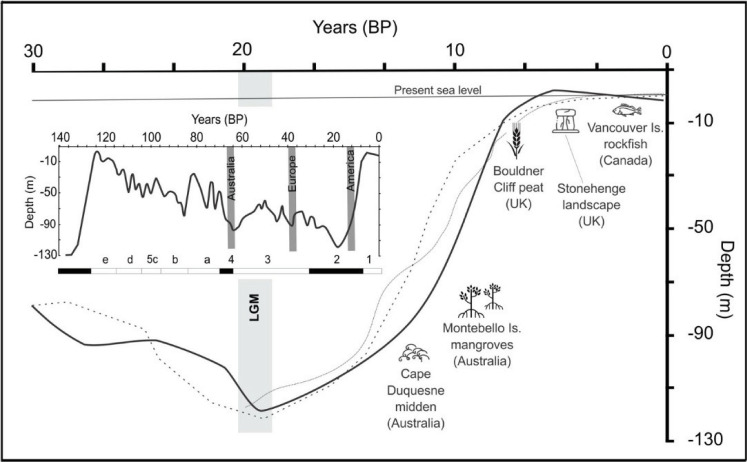
Global (solid lines) and relative (dashed lines) sea-level curves for 30 ka to present, showing the Last Glacial Maximum (LGM) lowstand, and examples of dated cultural events as determined from sedaDNA (refer to text for details). Inset shows the global sea-level curve from 120 ka with isotopic stages marked and highlighting the earliest known occupation ages in Australia, Europe and America (grey bar; after [15]).

The relationship between human communities and the land and sea will always be dynamic, shaped by climatic fluctuations, sea-level changes and biogeographical shifts. One of the most significant changes Indigenous people in Australia would have experienced is the sea-level rise from a low of −120 m during the last Ice Age, around 21 000 years ago to its present level. As the temperature increased, vast areas of Australia’s inner continental shelf (approx. 2 million square kilometres) and consequently a significant part of human–environmental history were submerged by the post-glacial sea-level rise (figure 1). These changes in sea-level and climate altered landscapes and affected the distribution of plant and animal species, leading to changes in biogeography, biodiversity and hence human resources ([16] and references therein; see also [17,18]).

In recent years, the study of sedimentary ancient DNA (sedaDNA) has expanded our understanding of past ecosystems. This innovative approach provides detailed insights into past flora, fauna and microbial communities and human activities through the analysis of genetic material preserved in sedimentary matrices [19–25]. SedaDNA forms through the gradual accumulation and preservation of genetic material from various organisms, including plants, animals and microorganisms. These organisms shed cellular debris, faeces, pollen and other biological remnants into their environment over time, which then become integrated into sediment layers [20]. The study of human–environmental interactions has been boosted by the use of sedaDNA, providing insights into how past human populations interacted with their environments [23,26–28]. By reconstructing past biodiversity, climate conditions and anthropogenic impacts, sedaDNA analysis has the potential to offer a detailed and nuanced understanding of how early people adapted to, altered and managed their landscapes. However, its application in Australia is still nascent, perhaps challenged by the country’s diverse, but often hot and harsh climates that accelerate DNA degradation.

SedaDNA has distinct advantages and disadvantages compared to traditional proxies like pollen and macrofossils. Its primary advantage is high taxonomic resolution, allowing the detection of a wider range of species, including those not typically preserved in the fossil record. This improves vegetation reconstructions, particularly where macrofossils are scarce [29,30]. Additionally, sedaDNA captures genetic information from diverse organisms such as bacteria, phytoplankton and zooplankton, aiding in reconstructing past ecosystems and biodiversity changes [31]. Challenges include potential contamination from modern DNA complicating results interpretation [32], and preservation issues influenced by taphonomic processes affecting DNA quality and quantity [33]. Metabarcoding biases may also lead to underrepresentation of species with shared haplotypes [34]. Thus, careful data interpretation and robust authentication methods are essential to distinguish ancient from modern DNA [32]. Combining sedaDNA with other proxies, such as pollen analysis and stable isotopes, enhances understanding of past environments. While pollen analysis offers regional vegetation insights, sedaDNA better reflects local biodiversity and fine-scale ecological changes [29,30]. Integrating multiple proxies strengthens palaeoecological reconstructions, providing more accurate assessments of past climate and biodiversity shifts [35,36].

SedaDNA is best preserved in sediments with high clay and organic content, especially under cold, dry or anoxic conditions [31,37]. These conditions help protect DNA from degradation because of reduced microbial activity, limited oxygen exposure and minimized chemical reactions such as hydrolysis, oxidation and alkylation that would otherwise break down the DNA over time. Specifically, lake sediments with low electrical conductivity and pH and marine sediments rich in clay and organic matter offer optimal preservation [32,38–42]. In Australia, coastal and marine sediments also offer promise for the study of prehistoric occupation and changing biogeography during periods of sea-level change as well as pre- and post-colonization. This article investigates the potential of sedaDNA preserved in Australia’s coastal margins to shed light on aspects of past human–environmental interactions. We highlight the valuable insights that sedaDNA offers for understanding past ecological conditions in Australia and for fostering the development of a more sustainable biocultural landscape in the future.

## 2. Emerging potential of sedimentary ancient DNA analysis in Australia

While sedaDNA’s potential for reconstructing past environments and biodiversity is well-recognized [19,22,24,29,43], its application in Australia is still in its early stages. The few published studies to date (table 1) have focused on assessing DNA preservation in fossils and sediments from Kelly Hill Cave, Kangaroo Island, revealing excellent long-term preservation of ancient DNA, highlighting its potential for uncovering extinct Pleistocene species and comparing genetic biodiversity with mainland records [44]; exploring recent fire and ecosystem change from lake sediments on Kangaroo Island (Karti) in South Australia [51]; and identifying oceanographic shifts and associated changes in algal and planktonic protist assemblages over the last 9000 years and also in the historic period from marine sediments off Eastern Tasmania [52]. While globally, much of the published research on sedaDNA has focused on sub-polar environments, the studies mentioned above highlight the potential of this field in warmer climates, as evidenced by analogous research conducted at sites outside Australia [41,53–55]. Below, we discuss and explore the potential of sedaDNA in Australia, focusing on human activity, coastal connections and ecological histories, including mangrove changes in Northwestern Australia.

**Table 1.** Summary of peer-reviewed studies on sedaDNA conducted in Australia over the past 10 years. This table presents a curated selection of primary research and review articles focusing on sedaDNA recovered from a range of sediment types across Australian environments. The studies span coastal marine, estuarine, lacustrine and cave systems, highlighting advances in methodology, palaeoecological reconstructions and ethical considerations in sedaDNA research. Articles were identified using the search terms ‘Australia’, ‘sedimentary ancient DNA’, ‘sedaDNA’ and ‘ancient DNA’ in Google Scholar, Scopus and Web of Science.

title	reference	sediment type	location	summary
Thorough assessment of DNA preservation from fossil bone and sediments excavated from a late Pleistocene–Holocene cave deposit on Kangaroo Island, South Australia	Haouchar *et al.* [44]	cave (fossil deposit)	Kelly Hill Cave, Kangaroo Island, SA	demonstrated exceptional DNA preservation in an Australian cave (Kelly Hill, Kangaroo Island) with ancient DNA from sediments and fossils up to approximately 20 000 years old
Broadening the taxonomic scope of coral reef palaeoecological studies using ancient DNA	del Carmen Gomez Cabrera *et al*. [45]	marine (reef)	Pandora Reef, GBR, QLD	pioneered sedaDNA analysis of coral reef sediments to reveal reef biodiversity (including non-fossilizing taxa), augmenting traditional palaeoecological records
An optimized method for the extraction of ancient eukaryote DNA from marine sediments	Armbrecht *et al.* [46]	marine (coastal)	Maria Island, TAS	tested multiple extraction protocols on marine sediment cores; identified an optimal workflow that maximizes recovery of authentic sedaDNA fragments
Hybridisation capture allows DNA damage analysis of ancient marine eukaryotes	Armbrecht *et al.* [47]	marine (coastal)	Maria Island, TAS	applied hybrid capture to marine sedaDNA, enriching ancient eukaryotic DNA and enabling detection of damage patterns to authenticate Pleistocene-age sequences
Using sedimentary prokaryotic communities to assess historical changes in the Gippsland Lakes	Pérez *et al.* [48]	lake (coastal lagoon)	Gippsland Lakes, VIC (Lake King and Lake Victoria)	analysed ancient bacterial DNA from Gippsland Lakes sediment cores to reconstruct historical water quality changes (eutrophication and pollution) over the past two centuries
More than dirt: sedimentary ancient DNA and Indigenous Australia	Lewis *et al.* [49]	terrestrial (perspective)	Australia (Indigenous contexts)	opinion piece highlighting ethical considerations, cultural sensitivities and benefit-sharing when conducting sedaDNA research in Indigenous Australian contexts
Recovering sedimentary ancient DNA of harmful dinoflagellates accumulated over the last 9000 years off Eastern Tasmania, Australia	Armbrecht *et al.* [50]	marine (coastal)	Maria Island, TAS	reconstructed approximately 9 000 year history of harmful algal blooms off Tasmania using sedaDNA; found long-term presence of toxic dinoflagellates (Alexandrium, Gymnodinium) and a recent surge in Noctiluca

### (a) Human activity and coastal connections

While it is clear that sedaDNA has the potential to revolutionize the study of paleoenvironments [56], it can also offer direct evidence of early human presence and migration patterns [57–59]. Recent years have seen increased archaeological and geoarchaeological exploration of Australia’s submerged landscapes [16,60,61], along with theoretical modelling to understand how sea-level changes and environmental shifts may have impacted past landscapes during the Pleistocene (e.g. [62] and references therein). While direct evidence for human presence on the submerged landscapes of the continental shelf remains elusive, studies from South Australia [63,64], as well as other parts of the world ), demonstrate that sedaDNA from marine contexts can reconstruct paleoenvironments before and during periods of inundation.

Indigenous narratives from Australia often contain ancient recollections of rapid coastal transformations, such as sea-level rise and episodic submergence of landmasses [65]. These narratives are deeply intertwined with Indigenous oral traditions, including Dreaming Ancestor narratives and songlines, providing insights into the historical connections between Indigenous peoples and the changing landscapes of Australia [66]. Consequently, what lies below the water and the seabed is as much a component of the cultural present as it is of the cultural past and is part of what is termed Sea Country. Integrating traditional knowledge and multi-millennial oral histories into sedaDNA research about past landscapes holds significant potential, although this integration is best led by Indigenous Australians themselves [67].

The potential to use sedaDNA to identify early use of coastal and marine resources is also of great interest, especially given recent evidence of shellfish exploitation as early as 42 000 years ago in North-West Australia [68]. Notably, most well-documented shell middens in Australia date to within the last 5000 years [69], a period marked by increased coastal stabilization and sea-level changes that provided more predictable and abundant marine resources. However, in southwestern Australia, despite the proximity to the coastline, the material record of marine culture diminishes, leading to the perception of a lack of a ‘coastal’ economy [69,70]. Current assessments of coastal exploitation may be biased by differential preservation, sampling or unknown palaeogeography [70,71]. A systematic and forensic approach to analysing sedaDNA signatures in both onshore and offshore sedimentary records can help address these biases. Additionally, incorporating Indigenous oversight from communities with connections to the land where the sedaDNA originates can enhance the relevance, palaeo-context and accuracy of the research [72].

Analysing plant and animal DNA in archaeological sediments can potentially reveal the resources exploited by ancient humans, shedding light on coastal and marine exploitation in response to fluctuating sea levels ([47] and references therein; [73]). In Canada, for example, researchers used archaeogenetic data of rockfish species from sites dating back to 2500 years ago to explore past fishing practices in an area of the Pacific Northwest Coast [74]. Critically, this study combined Indigenous knowledge and archaeological data with marine protected area management to enhance modern fisheries conservation and marine planning efforts. A further advantage of sedaDNA in these coastal and marine contexts is its potential to identify species that might be rare or difficult to observe directly [75], along with broad taxonomic coverage that allows for more comprehensive biodiversity assessments. However, Handsley-Davis *et al.* [76] caution that indiscriminate use of whole-genome or shotgun sequencing in Indigenous contexts can raise several ethical issues. These include concerns about consent, as Indigenous communities may not be fully informed about the implications in case ancient human DNA is profiled from the sediments. Albeit this is very rarely the objective in sedaDNA studies this must be clearly communicated as genetic data can reveal sensitive information about individuals or groups, potentially leading to exploitation or misrepresentation. Finally, cultural sensitivity must be respected, as some Indigenous groups may have specific beliefs or restrictions related to the treatment of biological materials from their ancestors.

Briggs [77] emphasizes the importance of linking identifiable DNA with archaeological evidence and other proxy data. For example, at the Bouldnor Cliff site offshore southern England, sedaDNA provided an earlier chronology for the introduction of wheat, dating it 2000 years before its known appearance in mainland Britain and 400 years before proximate European sites [78]. Although there were questions about the dating and taxonomic identification of wheat, including the lack of macrofossils, the authors defended their findings but acknowledged the need for further evidence to determine the DNA’s source [79]. Despite the controversies, the Bouldnor Cliff study still points to the sedaDNA potential of submerged sites, especially those with organic-rich peat deposits, to preserve earlier evidence that might not be available on land. DNA preservation in peats can present challenges due to their typically acidic nature, which accelerates DNA degradation. Nonetheless, certain peat types can create conditions favourable for DNA preservation by reducing oxygen levels, thereby inhibiting microbial activity and slowing the breakdown of DNA [80].

### (b) Ecological histories

SedaDNA has proven to be an invaluable tool for reconstructing past ecosystems and detecting shifts in biodiversity. Notable examples include its use in understanding the extinction events of megafauna during the Pleistocene (e.g. [81,82]), tracking the introduction and spread of invasive species (e.g. [83,84]), tracking historic records of harmful algal blooms [85] and uncovering the historical composition of vegetation [54,86,87]. These are all issues relevant to Australia, with the extinction of the megafauna around 40 000 years ago an ongoing topic of debate regarding its ecological impact [10,11,88]. Moreover, sedaDNA holds significant potential for exploring the introduction of non-native species and their implications for ecosystems in both terrestrial and marine settings [89], a critical issue given the role of invasive species in extinctions [90]. By analysing sedaDNA, it has become possible to uncover genetic signatures of non-native species, track their historical introductions and how their distribution has shifted over time, and assess their impacts on local biodiversity, ecosystem structure and function. For example, sedimentary records combining faunal and floral sedaDNA, along with erosion indicators, demonstrated the rapid impact of rabbit introduction on island ecosystems in the sub-Antarctic, leading to significant vegetation changes and soil erosion [91]. These insights from sedaDNA analysis can inform predictions about the future impacts of non-native species on ecosystems. By revealing the turnover of native to non-native species over time, sedaDNA can help understand past dynamics and forecast potential future scenarios, which is crucial for conservation and management strategies.

In providing a biostratigraphic record, sedaDNA can also serve as a chronological proxy for ecological change [92–94]. In particular, it can provide vital biological information in contexts where macrofossils (e.g. [75,95]) or even microfossils (e.g. [87]) are rare or lacking. Alsos *et al*. [95], for example, were able to use sedaDNA to show the persistence of floral species on Svalbard through periods when the associated macrofossils were not preserved, and in doing so demonstrated resilience of the flora to specific climate changes over the Holocene. As a study by Herzschuh *et al*. [64] indicates, potential also exists to use sedaDNA from deep-sea cores as a proxy for vegetation dynamics of nearby continents and, through this, linkages between the terrestrial and marine carbon cycles. Included in their study were sediment cores off Australia that showed a transition from coastal submerged (mangrove) plants to terrestrial regional plants associated with a rise in sea level from the Glacial to the Holocene (see also [63]). A similar study by Wee *et al*. [96] showed how sedaDNA can also provide a more nuanced understanding of the shifting composition of mangrove taxa in response to environmental fluctuations, beyond simple expansion and contraction models, including estuarine position, mangrove geomorphic type and bio-geomorphological complexity. These studies demonstrate that sedaDNA analysis is not limited to reconstructing past ecosystems in cold climates but can also be effectively applied in sub-tropical and tropical settings. Given the ecological significance of mangroves in equatorial latitudes and their sensitivity to climatic shifts, sedaDNA research has broader implications for understanding mangrove evolution, restoration and management in Australia. However, as Wee *et al*. [96] emphasize, international collaboration and capacity building in mangrove eDNA metabarcoding are crucial for advancing this field.

The marine sedimentary record could also help bridge a critical knowledge gap in our understanding of the Last Glacial Maximum (LGM) around 21 000 years ago, especially given the sparse and inorganic nature of terrestrial sediments from this period [97]. Contradictory records exist regarding the environmental impacts of the LGM, with some arguing for widespread aridification and forest loss [98] and others suggesting a positive moisture balance that supported more stable human occupation [99]. These differing records likely reflect regional variations, which are still not well understood, and are further complicated by limited chronological control for this period [97]. Hence, sedaDNA and other proxy data from nearshore and inundated continental shelf sediments could provide new insights into the timing and nature of these environmental changes and help clarify the impacts of the LGM. In the same vein, sedaDNA in marine records could potentially provide long-term records of ecology and fire history on Australia’s mainland continent (cf. [51]), although research is still needed to understand DNA preservation and dispersal on different parts of the inner shelf (see also [63]). A key aspect in all these studies is marine and coastal sediment coring and mapping (see also [100]).

## 3. Method advancements and future directions for sedimentary ancient DNA research in Australia

DNA is an unstable molecule that degrades over time [101], making the preservation of sedaDNA particularly challenging due to its degraded and fragmented state, low abundance and susceptibility to contamination from modern DNA [39]. In Australia, these challenges are exacerbated by the country’s diverse environmental conditions, which affect DNA preservation. Hot regions may accelerate DNA degradation, while coastal and marine environments may offer better preservation but complicate distinguishing ancient DNA from modern DNA [43]. To address these issues and ensure the authenticity of the sedaDNA signal, strict contamination control and optimized extraction protocols are necessary [31,81,94]. This is further aided through advancements in DNA extraction, purification techniques, library preparation, high-throughput sequencing and bioinformatics. The following outlines some of these developments.

### (a) Optimized DNA extraction and purification techniques

The optimization of extraction techniques for sedaDNA studies is crucial for maximizing DNA recovery from complex sediment samples. Australian coastal sediments exhibit a diverse mineral composition, primarily comprising clay minerals (kaolinite, illite and smectite) and heavy minerals (ilmenite, zircon, rutile and magnetite), shaped by local geology, hydrodynamic conditions and sediment sources [102]. These minerals vary in their capacity to adsorb and protect DNA fragments, with clay minerals often forming strong bonds with DNA, potentially hindering its recovery. Conversely, heavy minerals may have a lower affinity for DNA, but their presence can complicate extraction procedures due to the need for differential processing [103]. Therefore, the choice of extraction technique significantly impacts the recovery of mineral-bound DNA fragments from sediments, as it must be tailored to effectively disrupt these bonds and release DNA while minimizing degradation and contamination. Effective optimization ensures that the maximum amount of high-quality DNA is retrieved, which is essential for accurate downstream analyses. Lysis buffers play a significant role in maximizing the recovery of these fragments, directly affecting the overall yield and integrity of the DNA [103]. Silica magnetic bead-based purification methods are widely utilized for sedaDNA isolation due to their high binding affinity for nucleic acids, allowing for efficient separation of DNA from other sample components and resulting in high-purity DNA extracts [103]. Additionally, the modification of DNA-binding buffers, such as the Dabney binding buffer, enhances the recovery of shorter DNA fragments. This buffer is particularly crucial in sedaDNA studies for efficiently extracting highly degraded DNA from sediments due to its enhanced binding capacity with a guanidine thiocyanate-based solution, which binds even the smallest DNA fragments [104].

### (b) Advancements in library preparation techniques and high-throughput sequencing

Advancements in library preparation techniques, such as hybridization capture, have significantly advanced sedaDNA research. The latter enables the precise enrichment of specific DNA sequences from complex sediment samples [31,43]. This method employs biotinylated RNA baits to selectively capture and enrich low-abundance, degraded DNA fragments, thereby greatly enhancing the sensitivity and specificity of DNA recovery. As a result, hybridization capture has improved the accuracy and detail in reconstructing past ecosystems and biodiversity [31,43]. In Australia, where sedimentary environments are complex and ecosystems are unique, this technique is especially valuable. For instance, it can allow for the enrichment of DNA from underrepresented or extinct species, such as animals (e.g. greater stick-nest rat; [105]) and plants (e.g. Murnong yam daisy; [106]), historically significant to Indigenous peoples.

High-throughput ‘shotgun’ sequencing (HTS) has further advanced sedaDNA research by facilitating the simultaneous sequencing of millions of DNA fragments, enabling comprehensive metagenomic data retrieval from sediment samples without the need for prior amplification [107]. The integration of HTS with hybridization capture has resulted in higher resolution studies, reduced costs and cross-contamination minimization and detection, thereby enhancing the overall quality and efficiency of sedaDNA analysis [47,107]. The preservation of aDNA in Australia is highly influenced by the continent’s wide range of climates and environmental factors. Factors such as site-specific microclimates and sediment characteristics play crucial roles in DNA degradation rates. In regions where aDNA is highly degraded, shotgun sequencing can be advantageous over metabarcoding and other PCR-based methods. Shotgun sequencing does not rely on the presence of longer DNA fragments necessary for primer binding, making it more effective for analysing fragmented aDNA. However, the efficacy of shotgun sequencing is contingent upon the availability of comprehensive reference genomes to accurately map the sequenced data. Currently, there is a limited representation of Australian species in genomic databases, which poses challenges for precise taxonomic identification. Ongoing initiatives aim to expand these genomic resources (e.g. National Biodiversity DNA Library), thereby enhancing the utility of shotgun sequencing for Australian aDNA studies. It is important to note that metabarcoding has been successfully employed in various Australian studies. This method allows for the identification of multiple taxa within environmental samples and has proven effective in contexts where sufficient DNA fragment length is preserved. Therefore, the choice between shotgun sequencing and metabarcoding should be informed by the specific conditions of each site and the research objectives.

### (c) Bioinformatic techniques

Advancements in bioinformatics, especially in molecular authentication, have transformed ancient DNA research by improving the analysis and validation of ancient DNA samples. Tools like MapDamage are essential for verifying the authenticity of ancient DNA by identifying unique damage patterns typical of ancient specimens, helping to distinguish genuine ancient DNA from modern contaminants [108]. These tools have become essential in ancient DNA studies of humans [109,110] and megafauna [111,112], where authentication is critical for reliable outcomes. The use of MapDamage and similar bioinformatic packages has broadened the range of samples that can be confidently analysed, thereby enhancing our understanding of ancient ecosystems and biological processes [31]. Additionally, new tools like MetaDamage and PyDamage have been developed to analyse post-mortem damage in sedaDNA on a metagenomic scale and to automate damage identification in ancient DNA assembly [33,113]. Moreover, bioinformatic pipelines such as EAGER and GenErode offer comprehensive features for preprocessing, mapping, authenticating and assessing ancient DNA quality, aiding in ancient genome reconstruction and the study of genome erosion in endangered and extinct species [114,115]. All these pipelines are specifically designed to handle ancient DNA data, ensuring accurate and reliable analysis.

### (d) Multi-proxy approach

Integrating sedaDNA analysis with more traditional sedimentary proxies like pollen, lipid biomarkers, stable isotopes, geochemical markers, microfossils and macrofossils enhances the accuracy and depth of palaeoenvironmental research. Such a multi-proxy approach provides crucial environmental contexts, and the validity of sedaDNA data can be assessed by checking for age-associated DNA damage patterns and ensuring DNA has not moved vertically between sediment layers. Once validated, sedaDNA can be compared with other environmental proxies, such as plant pollen (e.g. [36,64,116]) and animal (including diatoms and foraminifera) microfossils [47,78, to not only cross-check results but also provide a more comprehensive reconstruction of past flora and fauna. Macrofossil analysis, which studies visible plant and animal remains, adds context and validates genetic data particularly in archaeological contexts (e.g. [117]). In Australian contexts, the integration of these methodologies could provide insights into the historical ecological dynamics of the region, especially in relation to human impacts on the environment. For instance, the combination of sedaDNA with lipid biomarkers and macrofossil evidence could elucidate the dietary practices and settlement patterns of Indigenous populations, as well as their interactions with changing environmental conditions. Such an approach would not only enhance the understanding of past ecosystems but also inform contemporary conservation efforts by providing a historical baseline against which current ecological changes can be measured.

Similarly, geochemical proxies, such as elemental ratios and trace metals, enrich paleoenvironmental reconstructions when integrated with sedaDNA, offering data on past productivity and environmental conditions [30,36]. Stable isotope analysis of elements like carbon, nitrogen, oxygen and sulphur provides insights into past biogeochemical cycles, environmental conditions, trophic interactions and nutrient cycling [118,119]. Lipid biomarkers reveal past biota and environmental conditions through the analysis of cell membrane molecules, aiding in reconstructing ancient microbial communities and identifying combustion products like polyaromatic hydrocarbons (PAHs) linked to human activities [120,121]; the latter is of particular relevance to Australia’s fire history. Understanding Australia’s fire history is crucial, as fire has shaped the landscape and influenced the evolution of its flora and fauna. Indigenous Australians have long used fire as a land management tool, employing cultural burning to promote plant growth, manage wildlife and reduce the risk of uncontrolled wildfires (e.g. [122]).

Integrating geochemical proxies, such as lipid biomarkers and trace metals, with sedaDNA analysis enables researchers to reconstruct historical fire regimes and assess their ecological impacts. Lipid biomarkers, for example, provide evidence of past vegetation types and responses to fire (e.g. [123]), while compounds like PAHs reveal combustion processes, whether natural or human induced (e.g. [124,125]). Sediment core analysis of these biomarkers allows researchers to infer patterns of fire activity, including timing, frequency and intensity. Stable isotope analysis complements these methods by offering insights into past biogeochemical cycles and environmental conditions (e.g. [126]). Variations in carbon and nitrogen isotopes, for instance, can reflect shifts in vegetation and productivity linked to fire events [127]. Together, these approaches create a comprehensive picture of how fire has interacted with climate, vegetation and human activity over time.

The analysis of macrofossil assemblages has been instrumental in evaluating anthropogenic impacts on marine ecosystems by documenting temporal shifts in community composition. For instance, Bauder *et al*. [128] analysed foraminiferal assemblages from a sediment core at One Tree Reef in the Great Barrier Reef, identifying ecological changes associated with European colonization and subsequent industrialization in Australia. Similarly, Martinelli *et al*. [129] demonstrated that dead molluscan shell assemblages at One Tree Reef accurately reflect living communities, underscoring the reliability of subfossil records in reconstructing past ecosystems. These studies highlight the utility of macrofossil records in detecting human-induced ecological changes. By comparing historical and contemporary assemblages, researchers can infer the extent of anthropogenic disturbances and guide conservation efforts. For example, shifts in species composition and abundance in molluscan death assemblages have been linked to human activities such as overfishing and habitat modification. Integrating macrofossil analysis with sedaDNA has the potential to leverage the strengths of both methods, offering a more comprehensive understanding of historical biodiversity and ecological changes.

### (e) Incorporating traditional knowledge

In all the above, multidisciplinary approaches enhance the robustness of reconstructions and provide complementary lines of evidence to address complex questions about human–environment interactions in past and present landscapes. Collaboration with Indigenous Australians is critical not only regarding ethical considerations but also in terms of the responsibilities of Indigenous peoples in caring for the Country [67,72]. Integrating sedaDNA analysis into archaeological workflows has also yielded transformative insights into the human past [23], but as genomic research continues to develop, ethical concerns remain paramount. Of note are the distinctions between what is regarded as human or non-human, Indigenous or Western contexts in research involving water, landscapes and natural phenomena [72]. Involvement of Indigenous Australians and making the work meaningful to all stakeholders is key. A good example is the use of environmental DNA work to monitor biodiversity in southern Australia by Esperance Tjaltjraak Native Title Aboriginal Corporation, including pilot work exploring this potential in offshore contexts. As one senior elder, Uncle Henry Dabb, explains, ‘*Our ways are about reading the landscape - that’s our cultural science. This work is part of that—backing up our ways of Knowing Country*’.^1^ This is especially important given the Esperance region is one of Australia’s biodiversity hotspots [130]. Ultimately the key is braiding modern research approaches (including sedaDNA) with traditional ways of understanding the world around us.

## 4. Conclusion

The potential of sedaDNA to contribute significantly to coastal and marine studies in Australia is immense. This leading-edge method opens new pathways for reconstructing past environments and ecologies, offering crucial insights into historical biodiversity and ecosystem dynamics unique to the Australian context. SedaDNA, especially when combined with other proxies, can provide detailed information on past vegetation and marine and terrestrial fauna. By mapping out such changes in the biodiversity over time, sedaDNA can also capture human presence and potentially illuminate migration patterns of early Indigenous Australians. Through the analysis of sedaDNA, researchers can gain a nuanced understanding of how Australia’s ecosystems have evolved over time in response to climatic shifts, human activities and other environmental factors. Moreover, sedaDNA enhances the interpretation of historical ecological data specific to Australian landscapes and marine environments, thereby improving predictions of future environmental changes and informing targeted conservation efforts. The diverse and unique environments of Australia make sedaDNA an especially powerful tool for studying the continent’s rich ecological and cultural history.

**Figure 2.**
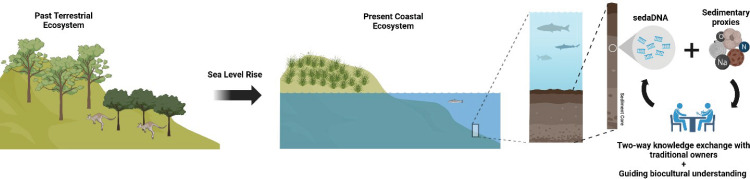
Application of sedaDNA to coastal margins that have been inundated due to sea-level change. In Australia, sedaDNA along with other sedimentary proxies can be integrated into a two-way knowledge exchange with traditional owners and aid in guiding biocultural understanding. Created with BioRender.com.

In Australia, integrating sedaDNA with other sedimentary proxies offers an opportunity for meaningful two-way knowledge exchange with traditional owners, fostering biocultural understanding (figure 2). Future research should prioritize understanding how climatic changes have shaped the composition of Australia’s coastal ecosystems. This can be achieved by analysing sediment cores using sedaDNA, radiocarbon dating and other paleoclimate proxies to reconstruct past environments. Key research questions include: How have climatic changes shaped the composition of marine and coastal ecosystems in Australia over time? What are the historical patterns of biodiversity in Australian marine and coastal environments, and how have they been affected by human activities? How did Indigenous Australians interact with coastal and marine environments, and what lessons do these interactions offer for current conservation efforts? What are the long-term impacts of human activities, such as fishing and land-use changes, on marine biodiversity in Australia? How has coastal vegetation responded to changing tidal regimes and sea-level shifts? Addressing these questions through interdisciplinary approaches will deepen our understanding of past ecosystems and inform sustainable management strategies for Australia’s unique coastal and marine environments.

## Data Availability

This article does not contain any new data.

## Footnotes

^1^
https://www.niaa.gov.au/news-and-media/two-way-cultural-knowledge-systems-and-new-edna-techniques-success#:~:text = Uncle%20Henry%20Dabb%2C%20Senior%20Cultural,Knowing%20Country%2C'%20he%20explains.
